# Large‐scale distribution patterns of mangrove nematodes: A global meta‐analysis

**DOI:** 10.1002/ece3.3982

**Published:** 2018-04-16

**Authors:** Marco C. Brustolin, Ivan Nagelkerken, Gustavo Fonseca

**Affiliations:** ^1^ Centre of Sea Studies Federal University of Paraná Pontal do Paraná Brazil; ^2^ Department of Marine Sciences Federal University of São Paulo Santos Brazil; ^3^ Southern Seas Ecology Laboratories School of Biological Sciences and The Environment Institute The University of Adelaide Adelaide SA Australia

**Keywords:** biodiversity, free‐living marine nematodes, landscape structure, macroecology, meiofauna, spatial distribution

## Abstract

Mangroves harbor diverse invertebrate communities, suggesting that macroecological distribution patterns of habitat‐forming foundation species drive the associated faunal distribution. Whether these are driven by mangrove biogeography is still ambiguous. For small‐bodied taxa, local factors and landscape metrics might be as important as macroecology. We performed a meta‐analysis to address the following questions: (1) can richness of mangrove trees explain macroecological patterns of nematode richness? and (2) do local landscape attributes have equal or higher importance than biogeography in structuring nematode richness? Mangrove areas of Caribbean‐Southwest Atlantic, Western Indian, Central Indo‐Pacific, and Southwest Pacific biogeographic regions. We used random‐effects meta‐analyses based on natural logarithm of the response ratio (lnRR) to assess the importance of macroecology (i.e., biogeographic regions, latitude, longitude), local factors (i.e., aboveground mangrove biomass and tree richness), and landscape metrics (forest area and shape) in structuring nematode richness from 34 mangroves sites around the world. Latitude, mangrove forest area, and forest shape index explained 19% of the heterogeneity across studies. Richness was higher at low latitudes, closer to the equator. At local scales, richness increased slightly with landscape complexity and decreased with forest shape index. Our results contrast with biogeographic diversity patterns of mangrove‐associated taxa. Global‐scale nematode diversity may have evolved independently of mangrove tree richness, and diversity of small‐bodied metazoans is probably more closely driven by latitude and associated climates, rather than local, landscape, or global biogeographic patterns.

## 1 INTRODUCTION

The hot spot of tropical marine biodiversity observed in the Indo‐West Pacific (IWP) region is a well‐recognized macroecological pattern described for many coastal and marine plant and animal species, although neither the processes nor the mechanisms responsible for this are well understood (Bowen, Rocha, Toonen, & Karl, 2013). Studies on mangroves and associated macroinvertebrate species corroborate this pattern, as well as studies on coral reefs and their associated fish and foraminifera communities (Bellwood & Meyer, 2009; Ellison, 2008; Gaither & Rocha, 2013; Renema et al., 2008). The epicenter of diversity in the IWP has been traditionally associated with Pleistocene sea level changes and the geographical complexity of the area (Bellwood, Hughes, Connolly, & Tanner, 2005), but molecular and fossil evidence from a range of taxa contradicts this notion and points to the presence of lineages from the Miocene, being much older than previously thought (Renema et al., 2008). Alternative hypotheses for its high biodiversity are that the IWP region may act either as a center of origin, overlap, or accumulation (Bellwood & Meyer, 2009; Bowen et al., 2013). Biodiversity hot spots have moved across almost half the globe over the past 50 million years with the timing and locations of their epicenter occurrences coinciding with major tectonic events (Leprieur et al., 2016; Renema et al., 2008). In this case, biodiversity hot spots are a product of ecological processes operating over geological timescales.

In relation to mangroves, one hypothesis is that they evolved around the Tethys Sea during the Late Cretaceous, and regional species diversity resulted from in situ diversification after continental drift (Ellison, Farnsworth, & Merkt, 1999). Mangrove tree species are uniquely adapted to tropical and subtropical coasts, and although they have a relatively low number of species, mangrove forests provide at least US $1.6 billion each year in ecosystem services supporting coastal livelihoods worldwide (Polidoro et al., 2010). Globally, mangrove forests are declining rapidly as they are cleared for coastal development, aquaculture and logged for timber and fuel production (Ellison, 2008; Hutchison, Manica, Swetnam, Balmford, & Spalding, 2014). This extensive habitat loss and fragmentation is generating extinctions and shifts in biodiversity with impacts on ecosystem functions and services (Snelgrove, Thrush, Wall, & Norkko, 2014; Thrush, Halliday, Hewitt, & Lohrer, 2008).

Little is known about the effects of mangrove forest area on local and regional populations of mangrove species and its associated fauna and flora (Nagelkerken et al., 2008; Polidoro et al., 2010). The aerial roots of mangroves partly stabilize the environment and provide a substratum on which many species of plants and animals live, while their leaf litter is transformed into detritus through herbivory, supporting complexes food webs (Nagelkerken et al., 2008; Somerfield, Gee, & Aryuthaka, 1998). The presence of mangrove pneumatophores increases algal retention, and therefore the density and diversity of associated meio‐ and macroinvertebrates (Bishop, Byers, Marcek, & Gribben, 2012; Gwyther & Fairweather, 2005). Hence, ecosystem engineering, facilitation cascades, and niche construction may have had an important role in generating and maintaining biodiversity of associated fauna at evolutionary timescales (Erwin, 2008). However, mangrove deforestation may result in biodiversity losses (Ellison, 2008; Polidoro et al., 2010) and change the biomass size spectrum of meiofaunal communities, for example, favoring small‐bodied nonselective deposit feeders above less generalist functional groups and species (Sabeel & Vanreusel, 2015). Generally, nematodes are the most abundant and diverse meiofaunal group inhabiting marine sediments. They play an important role in the remineralization of organic matter, and because they feed on a wide range of food items and have high functional diversity, they act as a critical link between microorganisms and higher food‐web levels (Hamels, Moens, Muylaert, & Vyverman, 2001; Pinto et al., 2013). Mangrove interstitial fauna is tightly associated with sedimentary microniches (Alongi, 1987; Pinto et al., 2013), and the higher richness in the IWP may be partly caused by variations in regional geomorphological complexity and habitat heterogeneity among ecoregions. Despite this, studies on the distribution of mangrove benthic fauna are mostly restricted to local‐scale patterns (Mokievsky, Tchesunov, Udalov, & Toan, 2011). On a global scale, it could be hypothesized that the longitudinal and latitudinal gradients in mangrove tree richness will drive richness of the associated fauna. Particularly for estuarine nematodes, it has been suggested that global patterns are better explained by the moderate endemicity hypothesis (MEH), which suggests that nematodes are dispersion‐limited and their distribution is influenced by local and regional environmental conditions rather than a homogeneous distribution across the globe (Fonseca & Netto, 2015; Fontaneto, 2011).

Estimates of the number of meiofauna species inhabiting mangrove sediments vary widely and come from a heterogeneous set of mangrove habitat types (Nagelkerken et al., 2008). Mangroves can differ in their size and shape, and mangroves with a different perimeter‐to‐area ratio might affect the structure of associated fauna differently (Boström, Pittman, Simenstad, & Kneib, 2011). Furthermore, mangroves with a similar total surface area and shape can differ in their aboveground biomass per unit of area, as well as their flow through adjacent habitats (Boström et al., 2011). However, whether such landscape heterogeneity explains heterogeneity in nematode richness within mangrove forests is still unknown.

We here address the following questions: (1) can species richness of mangrove trees from different ecoregions (i.e., Central Indo‐Pacific, Southwest Pacific, Western Indian, and Caribbean/Southwest Atlantic regions) explain large‐scale spatial patterns of nematode richness? and (2) do local landscape attributes have equal or more importance than biogeographic patterns in structuring nematode richness? To evaluate which factor is more important in determining nematode richness, a random‐effects meta‐analyses of published studies from around the world were performed.

## 2 METHODS

### 2.1 Data selection

**Figure 1:**
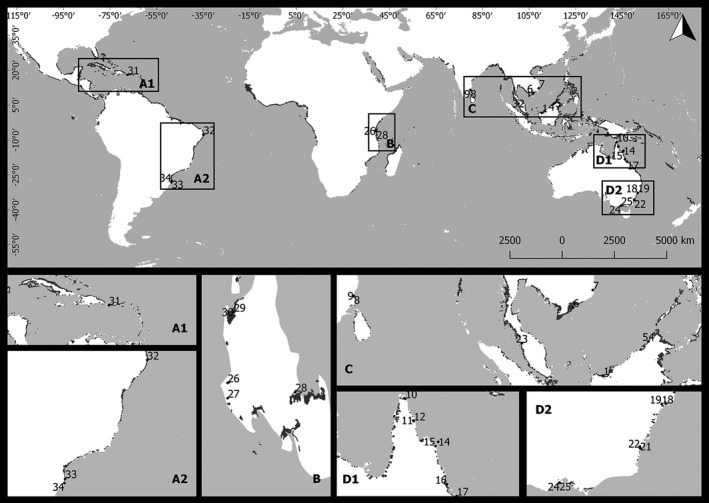
Global distribution of studies (*n* = 34) on mangrove nematode richness up to year 2016 in each marine biogeographic region: Caribbean‐Southwest Atlantic (A1 and A2); Western Indian (B); Central Indo‐Pacific (C); Southwest Pacific (D1 and D2)

The literature search was based on studies on nematode fauna from mangrove forests, published in journals indexed in Thomson Reuters' Web of Science, using the search strings “nematodes” + “mangrove.” We restricted our literature review to peer‐reviewed papers written in English. From the ∼5,950 published articles, 25 studies (covering 34 study sites) were included in the analyses (Figure 1). Mangrove forests within a study that were located in different estuaries were considered as different analytical units (sites). Only studies that reported average values accompanied by some measures of variance of nematode species richness were selected (Table S1). The average nematode richness per study was based on the arithmetic mean of samples from different sites. Sieve size and core volume were obtained from the same literature to evaluate the effects of sampling artifacts on nematode richness. The latitudinal distribution of mangrove tree richness was based on Ellison et al. (1999) and Ellison (2008). Ellison's datasets are composed of a presence/absence list of mangrove tree species across geographical coordinates. Mangrove tree richness was estimated as the total number of tree species occurring at the same degree of latitude and longitude for which diversity data of nematodes were available. Aboveground biomass, as well as spatial attributes of mangrove forests, like cover area and shape index, was obtained from modeled datasets (Giri et al., 2011; Hutchison et al., 2014). Aboveground mangrove biomass, mangrove forest cover area, and forest shape index of each mangrove forest included in the analyses were extracted from the original shapefiles through geostatistical tools available in the Quantum GIS software. Shape index was calculated as Perimeter/(2 * SquareRoot (PI * Area)) and measured using the Polygon Shape Indices module in SAGA‐GIS (Lang & Blaschke, 2007).

### 2.2 Meta‐analyses

In a meta‐analysis, results of independent studies are expressed as an index of effect. These effect size estimates are then combined across studies generating a summary of the outcomes. Also, subsets of studies can be examined separately to determine whether their outcomes differ or not (Hedges, Gurevitch, & Curtis, 1999). For each study, effect sizes of nematode species richness were calculated using the natural logarithm of the response ratio (lnRR). The log response ratio (lnRR) was used as an effect size because of its robustness to natural variability of ecological data and small sample sizes (Lajeunesse & Forbes, 2003). Traditionally, the effect size based on lnRR represents the ratio of the response variable measured in an experimental group to that of the control group (Hedges et al., 1999). In our study, the most distant site from the IWP hot spot (i.e., higher latitudes in the Atlantic Ocean) was used as the reference site, and species diversity of all other studies was compared relative to this site. In such a way, we evaluated the magnitude of change in diversity along an east/west spatial gradient.

Analyses were carried out using the R (version 3.3.1; R Development Core Team 2016) package “Metafor” (Viechtbauer, 2010). Weighted random‐effects models were carried out to calculate a summary effect size. Random‐effects analysis assumes that the true effect size differs between experiments, and the estimated summary effect is the mean of the effects observed across the studies. This meant that even if studies had a low weighting, the individual effect sizes from all studies could be incorporated into the summary effect (Borenstein, Hedges, Higgins, & Rothstein, 2009). Both the within‐study variance (inverse of the effect size variance) and the between‐study variance (σ^2^ pooled) were used to weight the studies. Therefore, studies with higher replication and/or lower variance were considered more precise and weighted accordingly (Gurevitch & Hedges, 1999; Hedges & Olkin, 1985). Between‐study variance was estimated using the DerSimonian–Laird method (DerSimonian & Laird, 1986). Statistical significance was attributed to each summary effect size by calculating a bias‐corrected 95% confidence interval (CI; Hedges & Olkin, 1985). If the confidence intervals do not overlap zero, then the effect size is considered significant (*p* < .05).

The total heterogeneity of a weighted mean effect size is represented by the *Q*_T_ statistic, which is a weighted sum of squares, comparable to the total sum of squares in an ANOVA. For each mean effect size, *Q*_T_ was calculated and tested against a χ^2^ distribution. A significant *Q*_T_ indicates that the variance among individual effect sizes is larger than expected by sampling error and that there may be an underlying structure to the data, and therefore, other explanatory variables should be tested (Borenstein et al., 2009). Biogeographic regions and sieve size were treated as categorical factors, while core volume, latitude, longitude, mangrove tree species richness, aboveground mangrove biomass, mangrove forest area, and forest shape index were treated as continuous variables. To evaluate which of those nine explanatory variables were more important for the observed underlying structure on nematode richness, a random‐effects metaregression, which is analogous to a multiple linear regression, was performed. For this model, total heterogeneity *Q*_T_ can be partitioned in the variance explained by the model (*Q*_M_) and the residual error variance not explained by the model (*Q*_E_). *Q*_M_ was tested against a χ^2^ distribution, and in this case, a significant *Q*_M_ indicates statistical differences in the relationship between effect sizes and predictor variables. Between‐study variance of lnRR was estimated using the restricted maximum likelihood (REML) method (Viechtbauer, 2010). The most parsimonious random‐/mixed‐effect model was chosen based on a step backward selection using Akaike information criteria (AIC). In addition, univariate random‐effect meta‐analyses exploring the individual relationship between the lnRR of nematode richness and all the nine explanatory variables are available in the Table S2.

### 2.3 Sensitivity analysis

Publication bias and between‐study heterogeneity for main effects were tested using Egger's regression test for funnel plot asymmetry (Egger, Davey Smith, Schneider, & Minder, 1997; Sterne & Egger, 2005). When a significant relationship between the observed outcomes (i.e., lnRR of nematode richness) and the standard error is detected, then this usually implies asymmetry in the funnel plot, which in turn may be an indication of publication bias. In the absence of bias and between‐study heterogeneity, the scatter plot will be due to sampling variation alone and the plot will resemble a symmetrical inverted funnel. If the heterogeneity fits with the model's assumptions, then the funnel plot will be symmetrical but with additional horizontal scatter. On the contrary, if heterogeneity is large enough to overwhelm the sampling error, then plots become cylindrical (Sterne et al., 2011). The output results from these analyses as well as funnel plots are available in Table S2 and Figure S2.

## 3 RESULTS

### 3.1 Large‐scale distribution pattern

**Figure 2:**
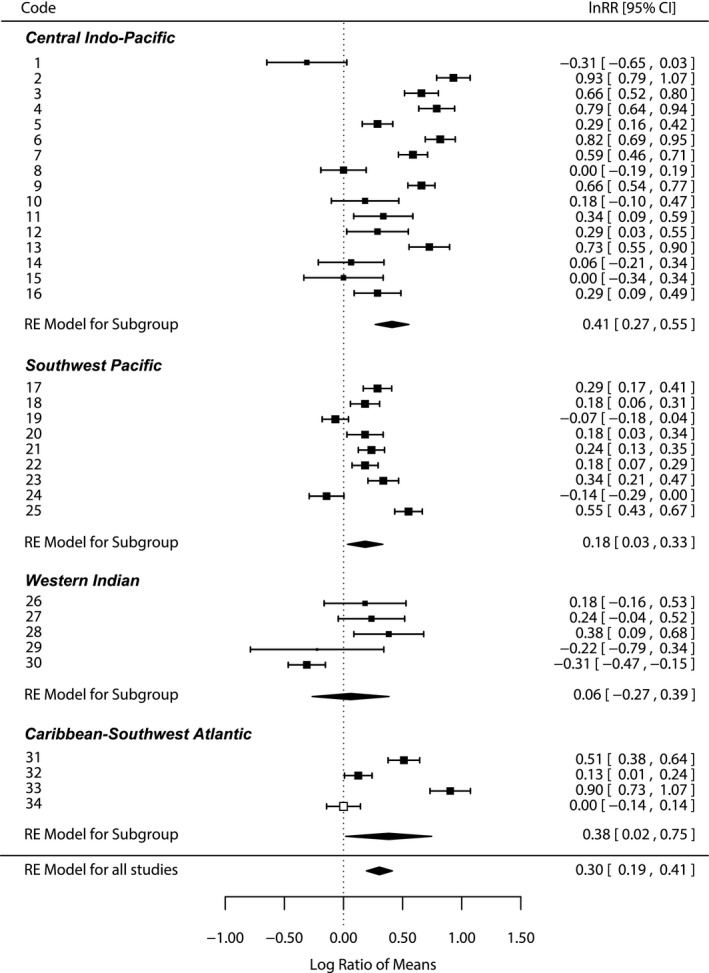
Natural logarithm of the response ratio (lnRR) of nematode richness. Horizontal black bars are 95% confidence intervals for effect sizes of each study included in the model; the size of the black squares represents the relative weight that each study had on the overall analysis. Open square indicates the farthest study along the east–west gradient, used as reference for the effect sizes estimation. Numbers in right column are average lnRRs with their respective lower and upper confidence intervals. Black diamonds are mean effect sizes for each ecoregion, and their length represents confidence intervals. 1‐Chen et al. (2012); 2‐Gee and Somerfield (1997); 3‐Somerfield et al. (1998); 4‐Shabdin and Othman (1999); 5‐Shabdin and Othman (2008); 6‐Xuan et al. (2007); 7‐Mokievsky et al. (2011); 8‐Chinnadurai and Fernando (2007); 9‐Ansari et al. (2014); 10‐11‐12‐15‐16‐Alongi (1987); 13‐14‐Decraemer and Coomans (1978); 17‐Alongi (1990); 18‐Hodda and Nicholas (1985); 19‐Hodda and Nicholas (1978); 20‐Nicholas et al. (1991); 21‐22‐Nicholas and Stewart (1993); 23‐Gwyther (2003); 24‐Gwyther and Fairweather (2002); 25‐Gwyther and Fairweather (2005); 26‐27‐28‐29‐Ólafsson (1995); 30‐Ólafsson et al. (2000); 31‐Torres‐Pratts and Schizas (2007); 32‐Pinto et al. (2013); 33‐Netto and Gallucci (2003); 34‐Fonseca and Netto (2006)

There was a significant heterogeneity (*Q*_T_) across studies (*p *<* *.001, Table S2). Therefore, the importance of several single explanatory variables was tested. At the biogeographic level, positive mean effect sizes in nematode richness were observed for the Central Indo‐Pacific, Southwest Pacific, and Caribbean‐Southwest Atlantic (Figure 2). Biogeographic regions explained alone 24.4% of the total heterogeneity across studies; however, the omnibus test for moderators indicated that there were no differences among biogeographic regions (*p *=* *.069, Table S2).

**Figure 3:**
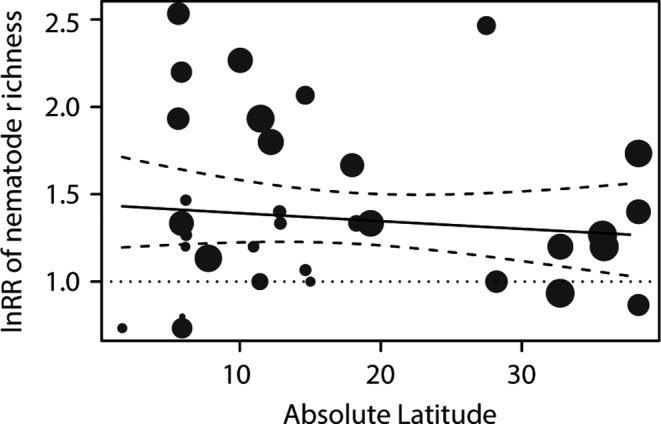
Scatterplot of the lnRR of nematode richness of the individual studies plotted against absolute latitude. The sizes of the dots are proportional to the inverse of the standard errors (i.e., studies with low internal variability are shown as larger dots). Solid line represents predicted values for a weighted regression line based on a mixed/random‐effects model (with corresponding 95% confidence intervals)

Among all the remaining descriptors, lnRR of nematode richness was only significantly correlated with latitude (Table S2, *R* ^2^ = 12.2%, *p *=* *.047), with higher richness occurring closer to the equator (Figure 3). In addition, the multiple metaregression model revealed latitude, but not biogeographic region as an important macroecological driver of the nematode richness (Table 1).

**Table 1:** Models, number of parameters, and values of adjusted Akaike information criteria (AICc), and difference between the model *i* and the best model (ΔAICc), for the alternative models (ΔAICc ≤ 2) explaining log response ratio outcomes from nematode richness of the summarized studies

Model	Parameters	*R* ^2^	AIC_c_	ΔAIC_c_
*lnrr ~ lat + area + shape + biomass + richness*	5	26.95	25.211	1.83
*lnrr ~ lat + area + shape + biomass*	4	25.72	23.707	0.33
*lnrr ~ lat + area + shape*	**3**	**19.35**	**23.377**	**0**
*lnrr ~ lat + shape*	2	14.31	24.622	1.24
*lnrr ~ lat*	1	12.22	24.050	0.67

Log response ratio outcomes (lnrr), absolute latitude (lat), total area of mangrove forest (area), shape index of mangrove (shape), total above ground biomass of mangrove (biomass), and number of mangrove tree species at each station (richness).

Bold values highlight the selected model.

### 3.2 Local landscape effects

The most parsimonious multiple metaregression model according to backward step AIC selection included absolute latitude, total mangrove forest area, and mangrove forest shape index as important variables, explaining 19.35% of the heterogeneity across studies (Table 2). Nevertheless, the two landscape attributes were marginally significant and were only responsible for 7.1% of total variance explained (see Figure S1). Aboveground tree biomass and richness of tree species had lower importance and did not explain a significant amount of the heterogeneity in nematode richness as verified by the higher AIC values observed when these variables were included in the multiple metaregression (Table 1). The Egger's regression test of the fitted model against its standard error indicated that there was a significant asymmetry in the funnel plot (*t* = −2.062, *df* = 29, *p *=* *.048) which may be an indication of a significant between‐study heterogeneity. Nevertheless, the funnel plot was symmetrical and horizontally dispersed, which indicates that the heterogeneity fits with the model's assumptions (Figure S2k).

**Table 2:** Summary of metaregression model with the respective values of correlation coefficients, standard errors (*SE*), *t*‐statistics, lower and upper confidence intervals for each selected explanatory variable. Asterisks represent significance of *p*‐values. Amount of variability across studies (*I* ^2^) and amount of variability across studies explained by the model (*R* ^2^) are stated as percentages. Degrees of freedom (*df*1 and *df*2), *F*‐statistic, and *p*‐value are from the omnibus test of moderators included in the model

	*I* ^2^	*R* ^2^	*df*1	*df*2	*F*	*p‐*Value
	92.57	19.35	3	30	2.197	.058
	**Coefficient**	***SE***	***t***	**Lower CI**	**Upper CI**	***p‐*** **Value**
Absolute latitude	0.0059	0.0032	1.843	0.0006	0.0125	.048
Mangrove area	−0.0015	0.0010	−1.528	−0.0035	0.0005	.060
Shape index	0.0095	0.0056	1.695	−0.0019	0.0209	.051

## 4 DISCUSSION

Macroecological distribution patterns of nematode species richness were not explained by the richness of mangrove trees. Heterogeneity of mangrove forests is important in nematode community assembly at the local (Pinto et al., 2013; Sabeel & Vanreusel, 2015) and regional scales (Fonseca & Netto, 2015). Yet, the present study showed that at larger spatial scales nematode richness is not directed related to mangrove tree richness. This pattern contrasts with that of crabs and littorinid gastropods, which are both strongly associated with mangrove tree richness (Ellison, 2008). Despite the lack of any relationship between nematode and mangrove richness, previous studies showed that nematode genus compositions differed among estuaries with and without mangroves (Fonseca & Netto, 2015). Habitat type is considered important in shaping benthic metacommunities from local to global scales (Nagelkerken et al., 2008; Pinto et al., 2013; Song et al., 2017). In this sense, mangrove tree richness may increase the number of habitat niches for macrofauna (Ellison, 2008). However, data on mangrove meiofauna are generally restricted to local studies that do not cover all habitat heterogeneities. Nematode diversity in the upper‐littoral zone where *Xylocarpus*,* Aegiceros*,* Heritiera*,* Acanthus*, and other mangroves tree species are distributed, are still poorly sampled and described. Therefore, the mangrove zones sampled in the studies that were included in our meta‐analysis do not reflect the entire mangrove floristic diversity or its full contribution toward structuring potential nematode richness.

Despite its relatively high explanatory power, there was no significant difference in nematode richness among biogeographic regions. Nematode species richness was not highest in the Central Indo‐Pacific, even though this biodiversity hot spot has already been described for several other marine coastal taxa, and the region has been considered as a center of origin, overlap, or accumulation of species (Bellwood & Meyer, 2009; Renema et al., 2008). Biodiversity hot spots such as the Central Indo‐Pacific harbor and export species, but can also accumulate biodiversity from adjacent areas. Both hot spots and peripheral ecosystems benefit from this biodiversity feedback (Bowen et al., 2013), and the complexity of the biogeographic area where a mangrove forest is located seems determinant for its tree and associated fauna richness (Ellison, 2008). This might not be necessarily true for nematode richness which seems more variable at smaller scales, probably due to their lower dispersal capabilities compared with macrofaunal invertebrates.

Latitude, rather than biogeographic region, was the main factor in structuring nematode richness at larger spatial scales. There was a significant correlation between nematode richness and latitude, with higher richness occurring at lower latitudes. The importance of latitude in structuring marine organisms has been reported for a variety of taxa and marine systems (Hillebrand, 2004). These latitudinal patterns of distribution might be related to temperature gradients, which suggest the roles of regional environmental and climatic factors in structuring nematode richness at large scales (Song et al., 2017).

Local landscape moderators had a secondary role in structuring nematode richness, with total mangrove area and forest shape index accounting for 7.1% of the total variance explained by our multivariate model. Shape index can be used as a proxy of landscape complexity. The weak but negative correlation between nematode richness and mangrove forest area as well as the positive correlation with shape complexity indicates that landscape structure can be a potential driver of spatial variation in nematode assemblages. In fact, the type of vegetation seems determining for nematode composition and structure in both marine and terrestrial environments (Fonseca & Netto, 2015; Song et al., 2017). However, whether the spatial heterogeneity within the same vegetation type influences nematode richness still needs better appreciation.

The fact that there was no correlation between nematode richness and aboveground biomass of mangrove forests was rather unexpected. Apparently, mangroves with distinct forest biomass can support a similar average nematode richness. The rapid generation time of nematodes compared with the time needed for leaf degradation may generate this decoupling between nematode diversity and mangrove leaf litter (Gwyther, 2003).

There were no significant effects of core volume or mesh size on average nematode richness. In our case, as methodological differences such as core volume or sieve size were not significant, the remaining heterogeneity might be either due to the relatively small number of observations or due to the intrinsic characteristics of studies that were not included in the model (e.g., environmental conditions at the local scale, differences in author's taxonomic accuracy, quality, and conservation of the sampled material). Despite the fact that meta‐analysis is robust to intermediate sample sizes (e.g., 20 < *k* < 50), and the confidence intervals generated are accurate (Hedges et al., 1999), incorporating more studies into future meta‐analyses will increase model robustness and accuracy. Also, sharing of detailed local‐scale data on abiotic factors rarely available in published studies (e.g., redox potential), as well the use of recurrent proxies such grain size and sorting, will enhance our understanding on how local and regional variations in environmental and biogeochemical conditions affect meiobenthic diversity and distribution.

In conclusion, our results contrast with biogeographic diversity patterns of highly associated mangrove taxa and species from other marine ecosystems. Global‐scale nematode diversity may have evolved independently of mangrove tree richness and is probably driven by regional and climatic factors. At local scales, nematode richness increased slightly with the complexity of the mangrove landscape. Overall, for small‐bodied taxa, latitude seems to overrule local factors and east–west biogeographic biodiversity patterns. This finding, therefore, has implications for patterns of meiofaunal species richness in a future world, where increasing ocean temperatures are driving range shifts of many species.

## CONFLICT OF INTEREST

Neither the manuscript nor any significant part of it is under consideration for publication elsewhere, nor has it appeared elsewhere in a manner that could be construed as a prior or duplicate publication of the same, or very similar, work. All of the undersigned authors participated actively in the study, and none has any potential conflict of interest. All of the authors have taken part in data analyses and interpretation. All of them have read and approved the manuscript in its present form and have agreed to its submission to Ecology and Evolution.

## AUTHORS CONTRIBUTION

MCB, IN, and GF conceived the ideas; MCB compiled and analyzed the data with the additional help of GF and IN; MCB led the writing with assistance from IN and GF.
