# Supplementary material for: Large‐scale distribution patterns of mangrove nematodes: A global meta‐analysis
Source: Ecol Evol. 2018 Apr 16;8(10):4734–42. doi: 10.1002/ece3.3982 (PMC5980601; doi:10.1002/ece3.3982)
Supplement: Supplementary file 1 [file ECE3-8-4734-s001.pdf]

Supporting information

Large-scale distribution patterns of mangrove nematodes – a global meta-analysis

Marco C. Brustolin - Ivan Nagelkerken - Gustavo Fonseca

Marco C. Brustolin (Corresponding author)

Centre of Sea Studies, Federal University of Paraná, Avenida Beira Mar s/n, Pontal do Sul, PO Box 61, Pontal do Paraná, PR 83255-976, Brazil

Department of Marine Sciences, Federal University of São Paulo, Rua Carvalho de Mendonça 144, Vila Belmiro, Santos, SP 11070-102, Brazil

e-mail: marcobrustolin@gmail.com
phone: 55 13 98132-7032/Fax: 55 13 3229-0100

Ivan Nagelkerken

Southern Seas Ecology Laboratories, School of Biological Sciences and The Environment Institute, The University of Adelaide, Adelaide, SA 5005, Australia

Gustavo Fonseca

Department of Marine Sciences, Federal University of São Paulo, Rua Carvalho de Mendonça 144, Vila Belmiro, Santos, SP 11070-102, Brazil

Tab. S1 List of references used in nematode richness data compilation. (Ref.) references; (Est.) Estuaries; (lat.) Latitude in UTM; (long.) Longitude in UTM; (core) core volume in cm^3; (mesh) mesh size in µm; (n) number of samples in each study; (st) number of stations in each study; (mean) mean nematode richness between stations; (S) standard deviation of nematode richness between stations; (S^2) variance; ($1/S^2$) inverse variance; (mean*$1/S^2$) weighted mean nematode richness.

Code	Ref.	Est.	lat.	long.	core (cm^3)	mesh	n	st	mean	S	S^2	$1/S^2$	mean*$1/S^2$
1	Chen et al., 2012	Teluk Awar	1.67	110.48	73.59	32	21	7	11	8.3	68.8	0.015	0.16
2	Gee and Somerfield, 1997	Sungai Merbok	5.64	100.45	-	63	40	17	38	12.2	149.1	0.007	0.25
3	Somerfield et al., 1998	Sungai Merbok	5.64	100.45	21	63	16	4	29	6.0	36.0	0.028	0.81
4	Shabdin and Othman, 1999	Lok Kawi beach	5.87	116.03	210	32	10	5	33	6.0	36.0	0.028	0.92
5	Shabdin and Othman, 2008	Lok Kawi beach	5.87	116.03	210	32	40	5	20	5.1	26.5	0.038	0.75
6	Xuan et al., 2007	Can Gio/Khe Nhan	10.04	106.77	96.16	38	12	4	34	4.6	21.3	0.047	1.59
7	Mokievsky et al., 2011	Be River estuary	12.20	109.18	28.26	40	9	3	27	2.9	8.3	0.120	3.28
8	Chinnadurai and Fernando, 2007	Coleroon/Vellar	11.44	79.78	70.65	63	20	5	15	5.6	31.0	0.032	0.48
9	Ansari et al., 2014	Vellar	11.48	79.77	24.53	53	72	24	29	6.8	45.6	0.022	0.64
10	Alongi, 1987	Escape river	-10.97	142.67	33	45	6	2	18	6.0	36.0	0.028	0.50
11	Alongi, 1987	Claudie river	-12.82	143.35	33	45	6	2	21	6.0	36.0	0.028	0.58
12	Alongi, 1987	Lockhart river	-12.88	143.38	33	45	6	2	20	6.0	36.0	0.028	0.56
13	Decraemer and Coomans, 1978	Lizard Island/mang A	-14.65	145.47	-	-	1	3	31	3.8	14.3	0.070	2.14
14	Decraemer and Coomans, 1978	Lizard Island/mang B	-14.65	145.47	-	-	1	7	16	5.6	31.1	0.032	0.51
15	Alongi, 1987	Morgan/McIvor	-15.01	145.23	33	45	6	2	15	6.0	36.0	0.028	0.42
16	Alongi, 1987	Missionary Bay	-18.27	146.22	33	45	12	4	20	6.0	36.0	0.028	0.56
17	Alongi, 1990	Chunda Bay	-19.28	147.05	33	45	72	2	20	5.7	32.0	0.031	0.63
18	Hodda and Nicholas, 1985	Hunter River	-32.73	151.68	29.44	50	84	14	18	6.0	35.5	0.028	0.50
19	Hodda and Nicholas, 1986	Hunter River	-32.73	151.68	29.44	50	140	28	14	3.9	15.2	0.066	0.95
20	Nicholas et al., 1991	Clyde River	-35.73	150.14	17.00	50	20	4	18	4.7	22.3	0.045	0.78
21	Nicholas and Stewart, 1993	Clyde River	-35.73	150.14	19.87	50	25	5	19	2.2	4.7	0.213	4.09
22	Nicholas and Stewart, 1993	Candlagan Creek	-35.84	150.17	19.87	50	25	5	18	2.0	4.0	0.250	4.60
23	Gwyther, 2003	Barwon River	-38.28	144.50	-	53	48	1	21	6.0	36.0	0.028	0.58
24	Gwyther and Fairweather, 2002	Barwon River	-38.28	144.50	-	53	162	3	13	9.0	80.3	0.012	0.16

Tab. S1	Continued												
**Code**	**Ref.**	**Est.**	**lat.**	**long.**	**core (cm^3)**	**mesh**	**n**	**st**	**mean**	**S**	**S^2**	**$1/S^2$**	**mean*$1/S^2$**
25	Gwyther and Fairweather, 2005	Barwon River	-38.27	144.47	-	53	150	3	26	9.4	87.9	0.011	0.30
26	Ólafsson, 1995	Maruhubi	-6.15	39.20	48	40	5	1	18	6.8	46.2	0.022	0.39
27	Ólafsson, 1995	Chukwani	-6.20	39.20	48	40	5	1	19	5.7	32.3	0.031	0.58
28	Ólafsson, 1995	Chwaka Bay	-6.18	39.42	48	40	5	1	22	7.0	48.5	0.021	0.45
29	Ólafsson, 1995	Muwanda	-5.92	39.22	48	40	5	1	12	7.6	57.7	0.017	0.20
30	Ólafsson et al., 2000	Muwanda	-5.92	39.22	42.5	40	35	7	11	4.0	15.9	0.063	0.67
31	Torres-Pratts and Schizas, 2007	Magueyes Island	17.97	-67.47	-	53	29	1	25	6.0	36.0	0.028	0.69
32	Pinto et al., 2013	Santa Cruz Channel	-7.77	-34.87	100	44	45	9	17	3.4	11.4	0.088	1.49
33	Netto and Gallucci, 2003	Ratones River	-27.48	-48.50	49.06	63	24	6	37	12.7	162.3	0.006	0.23
34	Fonseca and Netto, 2006	Laguna	-28.20	-48.63	31.4	63	60	15	15	6.0	36.0	0.028	0.42

Tab. S2 Omnibus test for heterogeneity (Q_T) and output results of the random-effects meta-analyses with the omnibus test (Q_M) for each single moderator tested and their respective *p*-values for Egger's regression test for funnel plot asymmetry. Relative amount of heterogeneity (i.e., between-study variance) in relation to the total unaccounted variability (I^2); Amount of variance (heterogeneity) accounted for by the moderators (R^2); Degrees of freedom (*df*); Test for residual heterogeneity (Q_E).

	**Heterogeneity**			**Mixed-effects**								**Egger's test**
	df	Q_T	*p*-value	I^2	R^2	*df*	Q_M	*p*-value	*df*	Q_E	*p*-value	*p*-value
Global Model	33	556.479	0.0001	-	-	-	-	-	-	-		0.0380
**Moderators**												
Marine Ecoregions	-	-		92.21	24.24	3	7.096	0.0689	30	384.975	0.001	0.0166
Longitude	-	-		94.23	0.00	1	0.109	0.7416	32	554.679	0.001	0.0382
Latitude	-	-		92.68	12.22	1	3.929	**0.0474**	32	437.341	0.001	0.0096
Mangrove richness	-	-		93.80	3.34	1	0.660	0.4166	32	516.222	0.001	0.0278
Biomass (AGB)	-	-		94.02	0.77	1	0.323	0.5698	32	535.489	0.001	0.0440
Mangrove area	-	-		93.87	2.59	1	0.012	0.9102	32	521.953	0.001	0.0335
Shape index	-	-		93.90	1.58	1	1.547	0.2135	32	524.585	0.001	0.0491
Sieve size	-	-		94.33	0.00	1	1.218	0.2698	30	529.553	0.001	0.0960
Core volume	-	-		93.57	0.00	1	0.448	0.5034	25	388.647	0.001	0.0736

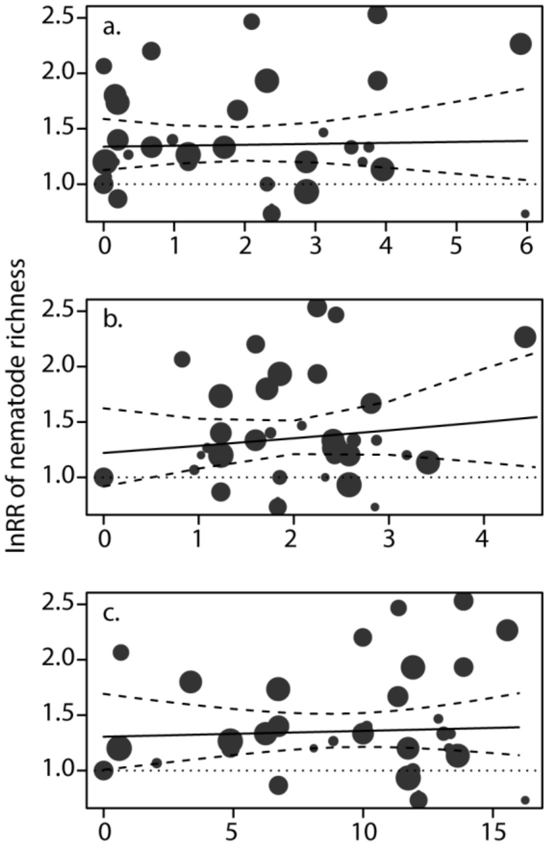

Fig. S1 Scatterplot of the lnRR of nematode richness of the individual studies plotted against the natural logarithm of mangrove forest area (a.), shape index (b.) and above-ground biomass (c.), respectively. Point sizes are drawn proportional to the inverse of the standard errors (i.e., more precise studies are shown as larger points). Solid line represents predicted values based on a mixed-effects model (with corresponding 95% confidence intervals).

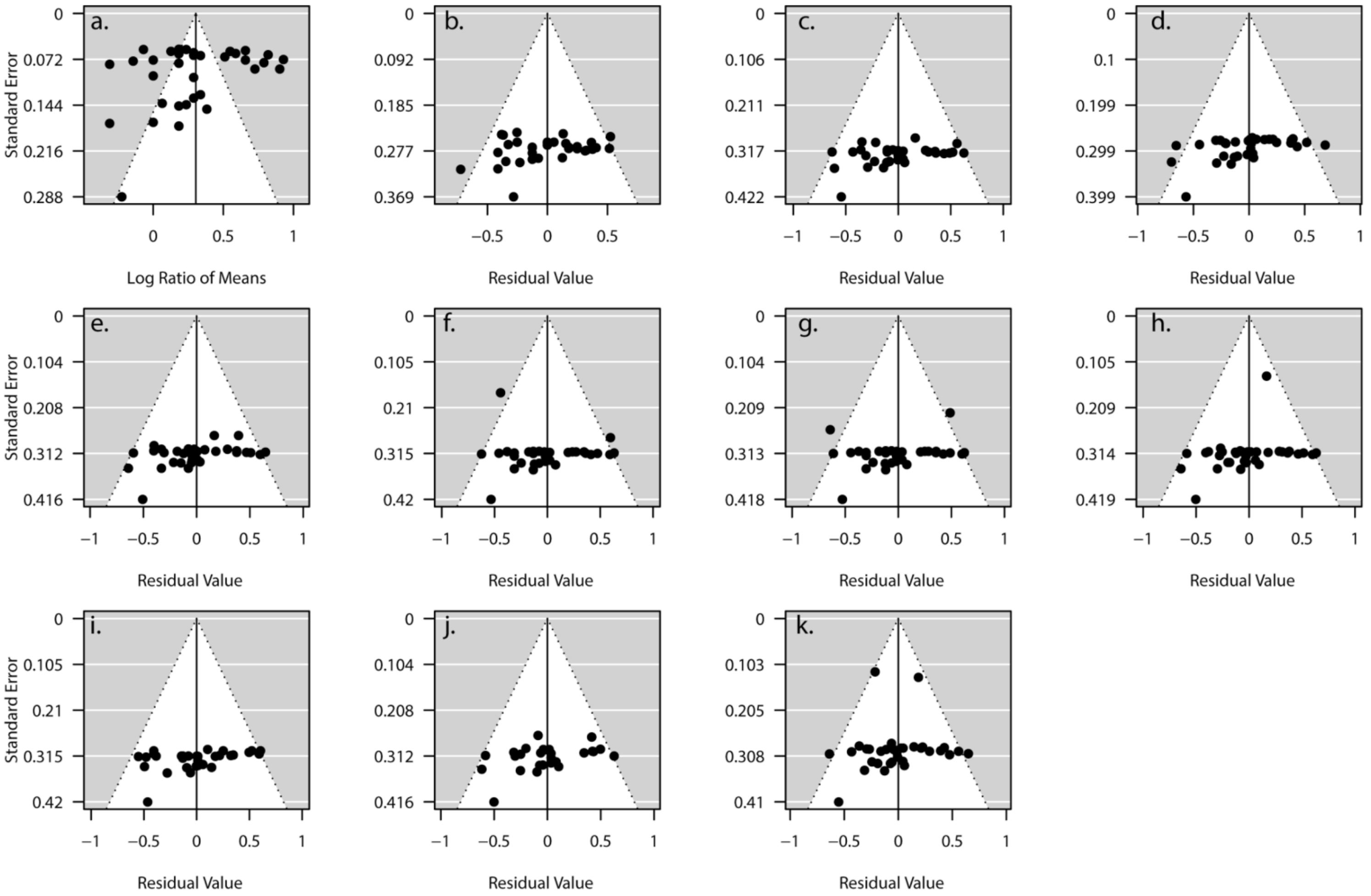

Fig. S2 Funnel plots of the effect estimates from individual studies against standard error of fitted univariate and multivariate random-effects meta-regression models. Outer dashed lines indicate the triangular region within which 95% of studies are expected to lie in the absence of both biases and heterogeneity. Metanalysis without any moderator (a.); marine ecoregions (b.); longitude (c.); absolute latitude (d.); mangrove richness (e.); above-ground biomass (f.); mangrove forest area (g.); shape index (h.); sieve size (i.); core volume (j.); multivariate meta-regression with absolute lat., cover area and shape index as continuous moderators (k.).
